# Genetic insights into unexplained infant jaundice: a study from northern Guangdong, China

**DOI:** 10.3389/fped.2026.1741740

**Published:** 2026-04-13

**Authors:** Fangjian Gao, Shuyan Li, Yali Chen, Yanting Zhou, Li Hu, Jianwu Qiu

**Affiliations:** 1 Guangdong Medical University, Zhanjiang, China; 2 Department of Neonatology, Yuebei People’s Hospital, Guangdong Medical University, Shaoguan, China; 3 Guangdong Province Critical Newborn Rescue Center (North Guangdong), Shaoguan, China

**Keywords:** gene, hyperbilirubinemia, infant, jaundice, neonate

## Abstract

**Objective:**

To explore the genetic factors contributing to unexplained infant jaundice and evaluate the significance of gene screening related to jaundice.

**Methods:**

Infants jaundice with unknown etiology attending the neonatology and pediatrics departments of Yuebei People's Hospital from January 2022 to July 2024 were selected as the subjects of this study. The exon regions of 161 jaundice-related genes were detected by targeted capture and high-throughput sequencing technology, and the results were statistically analyzed.

**Results:**

A total of 56 infants were included in the study, with 29 cases (51.8%) showing positive results. These cases involved six diseases: Gilbert syndrome in 7 cases (12.5%), sodium taurocholate co-transporting polypeptide (NTCP) deficiency in 8 cases (14.2%), glucose-6-phosphate dehydrogenase (G6PD) deficiency in 4 cases (7.1%), a combination of Gilbert syndrome and G6PD deficiency in 5 cases (8.9%), citrin deficiency combined with G6PD deficiency in 1 case (1.8%), Dubin-Johnson syndrome combined with Rotor syndrome in 1 case (1.8%), NTCP deficiency combined with G6PD deficiency in 2 cases (3.6%), and NTCP deficiency combined with Gilbert syndrome in 1 case (1.8%). Among the 56 infants, 55 cases (98.2%) had one or more gene mutation sites, with only 1 case (1.8%) showing no mutation sites. The five high-frequency mutation sites were the *UGT1A1* gene c.211G>A and c.-55_-54insAT sites, the *G6PD* gene c.1376G>T, c.871G>A, and c.1388G>A sites, and the *SLC10A1* gene c.800C>T site.

**Conclusion:**

Genetic factors significantly contribute to the development of infant jaundice of unknown etiology. Common pathogenic genes include the *UGT1A1*, *G6PD*, and *SLC10A1* genes, which have high-frequency mutation sites within the population. Conducting genetic screening for infants with jaundice of unknown etiology holds significant clinical importance.

## 1 Introduction

Jaundice is a common clinical issue in the neonatal period, characterized by an abnormal increase in serum bilirubin levels. It is estimated that over 80% of newborns will develop visible jaundice within the first week after birth, with approximately 10% requiring medical intervention (1). Hyperbilirubinemia not only affects the short-term health of newborns but can also impact their long-term neurodevelopment, especially when serum bilirubin levels exceed a certain threshold, potentially leading to severe complications such as kernicterus. Persistent hyperbilirubinemia can also have certain effects on various aspects of the body, including the immune system (2). The pathogenesis of hyperbilirubinemia involves multiple factors, among which genetic factors are particularly significant (3). Previous literature has primarily focused on individual genetic diseases associated with jaundice, and there are relatively few studies that have simultaneously investigated over 160 jaundice-related genes.

This study utilized Next-Generation Sequencing (NGS) technology to screen 161 genes associated with jaundice in infants experiencing unexplained jaundice. The aim was to thoroughly investigate the genetic factors contributing to infant jaundice and the mutation sites of these genes, thereby offering a robust theoretical foundation for gene screening in infants with jaundice.

## 2 Materials and methods

### 2.1 Study subjects and clinical data

Infants experiencing unexplained jaundice who were seen in the neonatology and pediatrics departments of Yuebei People's Hospital between January 2022 and July 2024 were selected as subjects for this study.

According to established pediatric guidelines, infants are classified based on their bilirubin characteristics: cholestatic jaundice is defined as direct bilirubin >17 μmol/L and a direct bilirubin/total bilirubin ratio >20 % (4).

The inclusion criteria were as follows: 1) Patient age: 0–1 year; 2) All patients met the diagnostic criteria for infant jaundice: In the neonatal period, based on the American neonatal bilirubin hour curve chart (1); jaundice lasting for an extended period (more than 2 weeks for full-term infants, more than 4 weeks for preterm infants) or recurring, with poor therapeutic response, and accompanied by persistent hyperbilirubinemia; a family history of adverse jaundice, such as blood exchange, kernicterus, etc.; 3) All subjects consented to the test voluntarily and provided signed informed consent from their parents.

Exclusion criteria included: infectious diseases, ABO incompatibility hemolytic disease, or jaundice caused by other definitive etiologies. This study was approved by the Ethics Committee of Yuebei People's Hospital (approval number: KY-2022-018), and informed consent was obtained from all parents of the infants.

### 2.2 Methods

#### 2.2.1 Candidate genes selecting

Through a literature review and expert consultation, we have identified 161 genes associated with over 200 hereditary diseases related to jaundice for inclusion in the current analysis. These diseases include those where jaundice is either the primary manifestation or a secondary symptom. The genes selected for sequencing primarily encompass *SLC10A1, UGT1A1, G6PD, SPTA1, PIEZO1, ATP7B, MYO5B, SMPD1, ABCC2, CFTR, SLC25A13, SLCO1B1, AGL, EPB41, HBB, NEK8, NOTCH2, SPTB*, among others. Information on genetic and hereditary diseases was drawn from authoritative databases like OMIM and Orphanet. Details of the comprehensive gene panel tested can be found in Supplementary Tables S1, S2.

#### 2.2.2 Gene sequencing

We employed high-throughput sequencing technology, achieving a depth surpassing 200×, to comprehensively encompass exonic regions and their adjacent intron-exon boundaries within a 50 bp flanking region. Peripheral blood samples (2 mL each) from the affected infants and their parents were collected using EDTA anticoagulant tubes. Genomic DNA was extracted, fragmented, and utilized for library construction and target capture sequencing. The results were analyzed, and Sanger sequencing was employed to further analyze and validate the positive variants, short deletions, or insertions identified by high-throughput sequencing within the family. The results were interpreted in accordance with the ACMG guidelines (5) to determine the pathogenicity of the variants. Infants diagnosed with genetic diseases were categorized as positive cases, whereas those not diagnosed with genetic diseases were categorized as negative cases.

### 2.3 Statistical methods

All statistical treatments were performed using SPSS Statistics 26.0 software. Normally distributed continuous data are presented as mean ± standard deviation, while non-normally distributed data are described by medians and quartiles. Normally distributed data was analyzed using the t-test, and non-normally distributed data was analyzed using the Wilcoxon rank sum test. Count data are expressed as numbers and percentages (%), and the differences were analyzed using the chi-square (χ^2^) test. Differences with *P* < 0.05 were considered significant.

## 3 Results

### 3.1 General conditions

This study enrolled a total of 56 infants with unexplained jaundice, including 35 males (62.5%) and 21 females (37.5%). The oldest confirmed case was 236 days old, while the youngest was 2 days old, with a median age of 40.50 days (interquartile range: 16.25–59.50 days). The minimum birth weight was 970 g, the maximum was 3,970 g, and the median birth weight was 2,955 g (interquartile range: 2,500–3,287 g). There were 13 low birth weight infants (23.21%) and 43 normal birth weight infants (76.79%). The minimum gestational age was 28 weeks, the maximum was 41 weeks, and the median gestational age was 38.1 weeks (interquartile range: 36.7–39.1 weeks). There were 14 preterm infants (25%) and 42 full-term infants (75%). The proportion of preterm infants was similar in the gene-positive and gene-negative groups (20.8% vs. 27.6%).

### 3.2 Disease status of infants with positive genetic sequencing

Among the 56 infants, 29 cases (51.8%) were diagnosed with genetic diseases, including 19 males (65.5%) and 10 females (34.5%). The positive rate in males was significantly higher than that in females, with a statistically significant difference (*P* < 0.01). The positive cases included six types of diseases: Gilbert syndrome (GS) in 7 cases (12.5%), Glucose-6-phosphate dehydrogenase (G6PD) deficiency in 4 cases (7.1%), sodium-taurocholate cotransporting polypeptide (NTCP) deficiency in 8 cases (14.2%), combined GS and G6PD deficiency in 5 cases (8.9%), combined Citrin deficiency and G6PD deficiency in 1 case (1.8%), combined Dubin-Johnson syndrome and Rotor syndrome in 1 case (1.8%), combined NTCP deficiency and G6PD deficiency in 2 cases (3.6%), and combined NTCP deficiency and GS in 1 case (1.8%). The genotypic details of these 29 infants are detailed in Table 1. Among these subjects, 26 infants carried at least one mutation site within these 161 genes but were not diagnosed as patients, while only 1 infant carried no mutations in these genes.

**Table 1:** Genotypes of 29 pediatric patients.

Disease	Genotypes	case load	gender	
			Male	Female
GS (7 cases)	UGT1A1 gene: c.211G>A (homozygote)	5	5	0
	UGT1A1 gene: c.211G>A and c.-55_-54insAT (compound heterozygote)	2	1	1
NTCP deficiency (8 cases)	SLC10A1 gene: c.800C>T(homozygote)	6	4	2
	SLC10A1 gene: c.800C>T and c.682_683del (compound heterozygote)	1	1	0
	SLC10A1 gene: c.800C>T and c.263T>C (compound heterozygote)	1	0	1
G6PD deficiency (4 cases)	G6PD gene: c.1,376G>T(heterozygote)	1	0	1
	G6PD gene: c.871G>A(hemizygote, heterozygote)	2	1	1
	G6PD gene: c.1388G>A(hemizygote)	1	1	0
Combined GS and G6PD deficiency (5 cases)	UGT1A1 gene: c.211G>A (homozygote);	1	1	0
	G6PD gene: c.1388G>A(hemizygote)			
	UGT1A1 gene: c.211G>A (homozygote);	1	1	0
	G6PD gene: c.1478G>A(hemizygote)			
	UGT1A1 gene: c.211G>A and c.686C>A(compound heterozygote);	1	1	0
	G6PD gene: c.871G>A(hemizygote)			
	UGT1A1 gene: c.211G>A and c.-55_-54insAT (compound heterozygote);	1	1	0
	G6PD gene: c.1376G>T(hemizygote)			
	UGT1A1 gene: c.211G>A (homozygote);	1	1	0
	G6PD gene: c.95A>G(hemizygote)			
Citrin deficiency and G6PD deficiency (1cases)	SLC25A13 gene: c.852_855delTATG(homozygote);	1	0	1
	G6PD gene: c.1376G>T(heterozygote)			
Dubin-Johnson syndrome and Rotor syndrome (1 cases)	ABCC2 gene: c.1462C>T (homozygote);	1	0	1
	SLCO1B3 gene: c.481+22insLINE(∼6.1 kb) (homozygote);			
	SLCO1B1 gene: c.1738C>T(heterozygote)			
NTCP deficiency and G6PD deficiency (2 cases)	SLC10A1 gene: c.800C>T(homozygote);	2	1	1
	G6PD gene: c.1376G>T(hemizygote, heterozygote)			
NTCP deficiency and GS (1 cases)	SLC10A1 gene: c.800C>T(homozygote)	1	0	1
	UGT1A1 gene: c.211G>A and c.-55_-54insAT (compound heterozygote)			

The median age at diagnosis was 40.0 days (interquartile range [IQR]: 9.5–59.0 days) for genetically confirmed positive cases, and 41.0 days (IQR: 27.0–60.0 days) for negative cases. There was no statistically significant difference between the two groups (*z* = −0.984, *P* = 0.325). No statistically significant differences were observed between the gene test-positive group (*n* = 29) and the negative group (*n* = 27) across all evaluated clinical laboratory indicators, including jaundice onset time, jaundice duration parameters, bilirubin fractions, liver function enzymes, and total bile acid levels (all *P* > 0.05). Infants in the cholestatic group had a significantly earlier age at jaundice onset than those in the non-cholestatic group [median 2.0 days (interquartile range, IQR: 1.0–2.0 days) vs. 2.0 days (IQR: 2.0–3.0 days); z = −2.136, *P* = 0.033]. For biochemical parameters, the cholestatic group had significantly higher levels of DBIL, TBA, and ALT (all *P* < 0.001), and significantly lower TIBL levels (*P* = 0.001) compared with the non-cholestatic group, with all the above differences reaching high statistical significance. No statistically significant differences were found between the two groups in jaundice duration, HB, IBIL, AST, or GGT levels (all *P* > 0.05). Further genetic diagnostic analysis confirmed the following hereditary jaundice subtypes in the cholestatic group: 5 cases of NTCP deficiency (SLC10A1 mutation), 5 cases of Gilbert syndrome, 2 cases of G6PD deficiency, 2 cases of NTCP deficiency combined with G6PD deficiency, 2 cases of Gilbert syndrome combined with G6PD deficiency, and 1 case of Dubin-Johnson syndrome. The overall genetic diagnostic rate was significantly higher in the cholestatic group than in the non-cholestatic group (Table 2).

**Table 2:** Comparative analysis of clinical biochemical parameters in different groups of pediatric patients.

Project name	Positive group	Negative group	*t/z* value	*p* value	Cholestatic group	Noncholestatic group	*t/z* value	*p* value
	(*n* = 29)	(*n* = 27)			(*n* = 29)	(*n* = 27)		
Onset of jaundice (d)	2.0 (1.0,2.5)	2.0 (1.0,2.0)	−0.492	0.623	2.0 (1.0,2.0)	2.0 (2.0,3.0)	−2.136	0.033*
Duration of jaundice (d)	34.5 (9.3,64.5)	37.0 (22.0,57.0)	−0.707	0.479	40.0 (14.0,72.5)	36.0 (20.5,45.3)	−0.885	0.376
HB (g/L)	127.19 ± 32.48	126.00 ± 32.17	0.138	0.891	123.12 ± 35.87	130.37 ± 27.53	−0.843	0.403
TIBL (μmol/L)	69.1 (41.6,198.2)	127.5 (90.7,221.3)	−1.697	0.09	52.0 (34.1,122.5)	179.1 (96.8,224.1)	−3.468	0.001**
DBIL (μmol/L)	26.8 (18.3,56.5)	22.1 (13.3,77.6)	−0.435	0.664	60.3 (40.6,144.7)	16.7 (12.4,20.7)	−5.911	0.000**
IBIL (μmol/L)	127.0 (49.6,197.4)	153.0 (80.6,256.1)	−1.049	0.294	127.0 (43.2,231.1)	166.3 (80.1,205.6)	−1	0.317
TBA (μmol/L)	141.6 (23.7,301.0)	28.7 (11.7,383.0)	−1.14	0.254	292.0 (108.6,425.0)	24.8 (9.6,51.0)	−4.468	0.000**
ALT(U/L)	21.5 (12.2,147.9)	26.7 (13.7,145.4)	−0.254	0.799	134.2 (16.1,197.1)	15.0 (12.0,24.4)	−3.337	0.001**
AST (U/L)	34.4 (22.5,52.2)	35.0 (21.7,121.4)	−0.771	0.441	32.3 (13.6,127.9)	35.0 (26.6,51.2)	−0.672	0.501
GGT (U/L)	136.0 (63.9,198.6)	110.6 (34.5,226.0)	−0.517	0.605	113.0 (29.7,245.1)	126.3 (73.4,174.0)	−0.746	0.456

Explanatory note:Hemoglobin: HB, Total Bilirubin; TIBL, Direct Bilirubin; DBIL, Indirect Bilirubin; IBIL, Total Bile acid; TBA, Glutamic-Pyruvic Transaminase; ALT, Glutamic-Oxalacetic Transaminase; AST, *γ*-Glutamyl Transpeptidase; GGT. *p < 0.05 **p < 0.01.

### 3.3 Analysis of gene variant carriage

Among the 56 infants, 55 cases (98.2%) exhibited one or more gene mutation sites, while only one case (1.8%) did not show any variation sites. Five variants were identified in the *UGT1A1* gene: c.211G>A, c.-55_-54insAT, c.1046C>T, c.1091C>T, and c.686C>A, with an overall carrier rate of 40.2%. The *SLC10A1* gene had three variants: c.800C>T, c.263T>C, and c.682_683del, with an overall carriage rate of 25.0%. The *G6PD* gene had five variants: c.1376G>T, c.871G>A, c.1388G>A, c.1478G>A, and c.95A>G, with an overall carriage rate of 10.7%.

**Figure 1:**
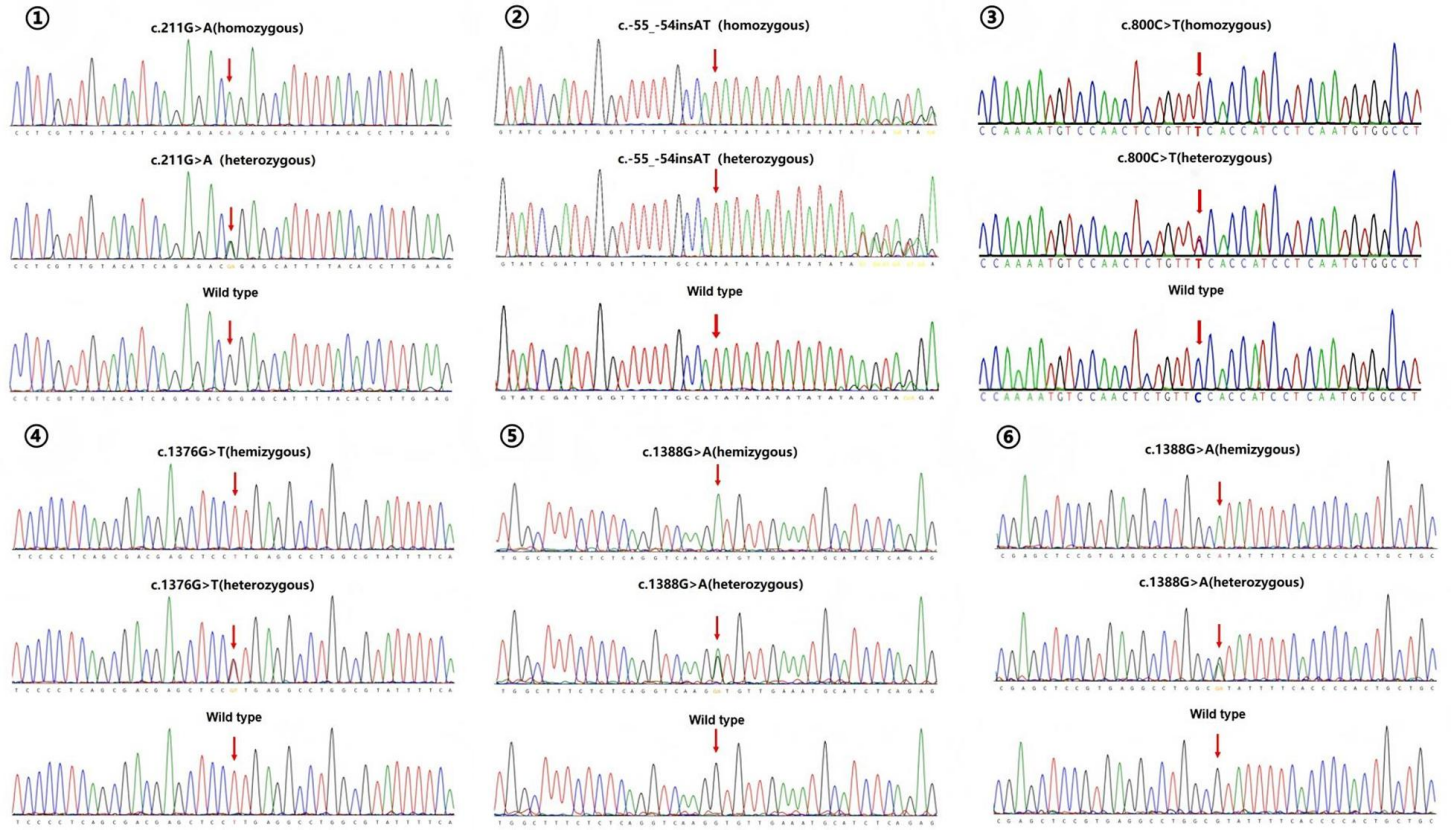
Sanger sequencing validation images of high-frequency mutation sites. ① UGT1A1 gene c.211G>A site; ② UGT1A1 gene c.-55_-54insAT site; ③ SLC10A1 gene c.800C>T site; ④ G6PD gene c.1376G>T site; ⑤ G6PD gene c.871G>A site; ⑥ G6PD gene c.1376G>T site.

Six high-frequency mutation sites (carriage rate >1%) were identified: the *UGT1A1* gene c.211G>A and c.-55_-54insAT sites, the *SLC10A1* gene c.800C>T site, and the *G6PD* gene c.1376G>T, c.871G>A, and c.1388G>A sites. The ClinVar database (https://www.ncbi.nlm.nih.gov/clinvar/) was queried to determine their pathogenic status, as shown in Table 3. The Sanger sequencing validation images of these six high-frequency mutation sites are shown in Figure 1.

**Table 3:** High-Frequency variant sites and allele frequencies in gene sequencing of infants with unexplained jaundice.

Gene	Variant sites	Amino acid changes	ClinVar state	Number of detections	Allele Frequencies (%)
UGT1A1	c.211G>A	p.Gly71Arg	Pathogenic (10); Likely pathogenic (4); Uncertain significance (3); Benign (1); Likely benign (3)	35	31.25
UGT1A1	c.-55_-54insAT	promoter variation	Pathogenic (12); Likely pathogenic (3); Uncertain significance (2); Benign (3)	7	6.25
SLC10A1	c.800C>T	p.Ser267Phe	Uncertain significance^1^; Benign (2)	26	23.21
G6PD	c.1376G>T	p.Arg45Leu	Likely pathogenic	5	4.46
G6PD	c.871G>A	p.Val291Met	Pathogenic, Likely pathogenic	3	2.68
G6PD	c.1388G>A	p.Arg463His	Pathogenic	2	1.79

## 4 Discussion and conclusion

The most significant harm of jaundice is the neurotoxicity of bilirubin, which is an extremely complex and serious issue. It can lead to kernicterus and may also cause bilirubin-induced neurologic dysfunction (BIND) (6). In addition, bilirubin may also have adverse effects on the kidneys, immune system, and endocrine system.

The etiology of infant jaundice and the factors that contribute to its delayed resolution are complex and multifaceted, encompassing physiological, genetic, environmental, and feeding factors. They may also be influenced by climatic elements such as temperature, rainfall, atmospheric pressure, cloud cover, and duration of sunshine (7). Genetic diseases and mutations in genes related to bilirubin metabolism (8) can both lead to elevated bilirubin levels, resulting in jaundice. Genetic factors are a significant cause of jaundice. For infants with unexplained jaundice, the possibility of genetic factors should be considered, and early genetic testing can assist in further clarifying the etiology in clinical practice (9). In this study, half of the infants with unexplained jaundice were found to have genetic causes, which is higher than the average positive rate in Guangdong Province (42%) (9). Six types of genetic diseases related to jaundice were identified in this study, with the most common ones being Gilbert syndrome, G6PD deficiency, and NTCP deficiency.

Gilbert syndrome is caused by reduced activity of uridine diphosphoglucuronate glucuronosyltransferase isoenzyme 1A1 (UGT1A1), the key enzyme involved in bilirubin metabolism, resulting in reduced bilirubin clearance by the liver and elevated serum bilirubin levels (10). Variations in the *UGT1A1* gene can cause varying degrees of quantitative reduction in UGT1A1 enzyme activity. The c.211G>A (p.Gly71Arg) variant, which diminishes enzyme function, is prevalent among Asian populations. Homozygous mutations can lower enzyme activity to (32.2 ± 1.6)% of the normal level, whereas heterozygous mutations reduce it to (60.2 ± 2.5)% of normal (11). The frequency of the *UGT1A1* gene c.211G>A variant in the jaundiced patient population of northern Guangdong is 31.25%, which is similar to the prevalence in Guangdong Province (9) at 32.3% and in Japanese patients (11) at 32.0%. The insertion variant in the TATA box (c.-55_-54insAT) is also a prevalent variant that decreases function. This pathogenic variant is situated in the promoter region upstream of the first exon of the *UGT1A1* gene. The repeated base pairs decrease the binding affinity of proteins to the TATA box, thereby reducing gene expression and UGT1A1 enzyme activity. Homozygous variants of c.-55_-54insAT can decrease UGT1A1 enzyme activity in liver tissue homogenates by 52%, while heterozygous mutations can reduce it by 37% (12).

The total population frequencies of both UGT1A1 gene variants c.211G>A and c.-55_-54insAT range from 0.5% to 5%; both have been detected in East Asian patients with Gilbert syndrome, which represent common polymorphic sites associated with the disease in these populations (13, 14). UGT1A1 gene c.211G>A variant: This variant resides within a critical structural domain of the protein, featuring amino acid sequences remarkably conserved across diverse species. Multiple computer-assisted analyses predict a moderate probability of impact on protein structure or function. Enzyme activity assays demonstrate that this variant significantly reduces UDP-glucuronosyltransferase activity, and subsequent functional experiments conclusively confirm its detrimental effect on enzyme function (15, 16). Furthermore, the frequency of this variant is markedly elevated in patient populations compared to normal cohorts (11, 17). UGT1A1 gene c.-55_-54insAT: This variant displays poor conservation of amino acid sequences across species and resides within the gene promoter region. It significantly reduces gene transcription efficiency, decreasing UDP-glucuronosyltransferase activity to approximately 30% of wild-type levels (18–20). Strikingly, when forming a compound heterozygous state with pathogenic variants, it causes a more pronounced enzyme deficiency, markedly elevating serum total bilirubin levels. According to ACMG scoring criteria, both variant sites are classified as Class 3 (variants of uncertain significance).

The frequency of the c.-55_-54insAT variant in the jaundiced patient population of northern Guangdong is 6.25%, which is lower than other reported rates of 9.0%–16.0% (21). Due to decreased enzyme activity, bilirubin clearance becomes challenging, leading to a delayed resolution of jaundice. Clinically, some infants with heterozygous mutations also experience a slow resolution of jaundice. Bilirubin-induced neurologic dysfunction (BIND) may also result from reduced UGT1A1 enzyme activity, which hampers bilirubin metabolism, causing the accumulation of unconjugated bilirubin in the blood. This can cross the blood-brain barrier and deposit in the basal ganglia, ultimately leading to neurologic damage (22). Gilbert syndrome generally does not require treatment. However, for infants with high bilirubin levels and persistent jaundice, low-dose phenobarbital can be used to induce UGT1A1 activity.

NTCP deficiency is a novel hereditary bile acid metabolic disorder caused by biallelic variants of the solute carrier family 10 member 1 (*SLC10A1*) gene. In 2015, Vaz et al. (23) reported the first case of NTCP deficiency and described the clinical manifestations of such patients. In 2017, Professor Song Yuanzong's team (24) reported on the youngest patient with NTCP deficiency and suggested that this condition could lead to neonatal hyperbilirubinemia. Subsequent studies have confirmed that 92.31% of patients with NTCP deficiency developed indirect hyperbilirubinemia in the neonatal period, experiencing a prolonged duration of jaundice resolution and normalization of high indirect bilirubin levels at approximately 2.5 months post-birth (25). The source of bilirubin in newborns is very abundant. Organic anion transporting polypeptides (OATPs) are the main transporters for the uptake of plasma bilirubin other than bile acids. In NTCP deficiency disease, hypercholanemia competitively inhibits the transport of bilirubin by OATPs, thereby causing neonatal hyperbilirubinemia (24). The c.800C>T variant in the *SLC10A1* gene is a high-frequency mutation site in northern Guangdong, with a carrier rate of 23.21% among jaundice patients, which is slightly lower than the carrier rate in Guangdong Province (9) of 29.6%.

The overall population frequency of the SLC10A1 gene c.800C>T variant in the gnomAD database falls below 5%. Amino acid sequence conservation in the variant's region is moderate across species. Multiple computational analyses strongly predict this variant will disrupt protein structure or function. Literature reports (26) indicate that while the variant does not impair estrone sulfate uptake, it nearly abolishes the protein's ability to uptake cholic acid and taurocholic acid. This variant has been identified in homozygous form in multiple clinical hypercholanemia cases (24, 27), and its frequency within the affected population significantly exceeds that in the general population (26). Moreover, proband family analyses often demonstrate co-segregation. Consequently, it is classified as a Class 1 pathogenic mutation under ACMG guidelines.

Currently, there is a lack of specific therapeutic drugs for NTCP deficiency disease, and symptomatic and supportive treatment is the main management approach. Hyperbilirubinemia in the neonatal period is primarily characterized by elevated indirect bilirubin, and conventional treatments such as phototherapy are effective. Some infants have deficiencies in zinc and vitamin D, which need to be corrected in a timely manner.

Glucose-6-phosphate dehydrogenase (G6PD) deficiency is an X-linked, incompletely dominant disorder affecting red blood cell enzymes and is a significant cause of pathological jaundice in newborns. Our study identified that the c.1376G>T, c.871G>A, and c.1388G>A loci in the *G6PD* gene are high-frequency mutation sites, which differ from those prevalent in Guangdong Province (9).

The overall frequencies of the three G6PD variants—c.1376G>T, c.871G>A, and c.1388G>A—remain remarkably low within the population, each falling below 0.5%. Critically, all three mutation sites reside within the protein's key functional domains, regions characterized by exceptionally high amino acid sequence conservation across diverse species. Multiple sophisticated computer-based analyses consistently predict a high to very high likelihood that these variants disrupt protein structure and function; these variants are typically inherited maternally. c.1376G>T: Definitive experimental studies confirm this mutation significantly reduces G6PD enzyme activity and severely diminishes its affinity for glucose-6-phosphate (28). Clinically, this variant has been documented in numerous cases of G6PD deficiency and has been previously designated as G6PD Canton. The alteration c.871G>A significantly compromises the enzyme's affinity for its substrate, as revealed by pertinent functional investigations (29, 30). Reported in clinical cases of G6PD deficiency, it has historically been named “G6PD Viangchan” and ranks among the most frequent variants causing G6PD deficiency across Southeast Asia (29, 31). c.1388G>A: Functional analyses demonstrate that this variant leads to markedly decreased G6PD enzyme activity and reduced affinity for glucose-6-phosphate (28). Observed in G6PD-deficient patients from multiple families, it represents a prevalent pathogenic mutation within Chinese populations and is known as “G6PD Kaiping,” “G6PD Anant,” and “G6PD Dhon,” among other names. Applying the stringent ACMG scoring criteria, all three G6PD gene variants—c.1376G>T, c.871G>A, and c.1388G>A—are classified as Class 1 pathogenic mutations.

This discrepancy may be attributed to the higher proportion of Hakka individuals in northern Guangdong, as c.1376G>T and c.1388G>A are known to be high-frequency mutation sites within the Hakka population (32–34). Children with G6PD deficiency should avoid drugs that cause oxidative stress. Those with high bilirubin levels can be treated with high-intensity blue light therapy.

The G6PD variants detected in this study were primarily associated with unconjugated bilirubinemia, despite the absence of clinically significant hemolysis in most cases. This pattern aligns with recent evidence indicating that G6PD deficiency primarily leads to neonatal jaundice through impaired conjugation of hepatobilirubin rather than hemolysis. The reduced availability of NADPH in hepatocytes compromises antioxidant defense and UGT1A1 enzyme function, resulting in functional jaundice (35). This study identified 10 infants simultaneously affected by two inherited jaundice disorders. Among them, 5 cases carried mutations in both G6PD and UGT1A1, while 2 cases carried mutations in both G6PD and SLC10A1. All exhibited severe or prolonged jaundice, suggesting that the coexistence of multiple jaundice disorders may lead to elevated bilirubin levels, thereby complicating the resolution of jaundice. This combination impairments both hepatic uptake/combination (UGT1A1) and antioxidant capacity (G6PD), explaining the severe phenotypes despite markedly normal hemoglobin levels. Clinically, these findings emphasize that even in the absence of overt hemolysis, G6PD deficiency should be considered a significant risk factor for neonatal hyperbilirubinemia (35). Additionally, the study revealed that the number of male infants was significantly higher than that of female infants. Previous research has shown that the incidence of jaundice in male newborns is higher than that in female newborns, which may be related to gender-specific physiological differences, although the exact mechanisms are not yet fully understood (36). The speculated reasons for this could be that males have a relatively higher bilirubin load per kilogram of body weight, or that androgenic steroids inhibit the enzymatic glucuronidation process, or a combination of both factors (37).

This study has certain limitations. It is known that certain non-genetic factors can influence the occurrence and severity of neonatal jaundice, including preterm birth and total parenteral nutrition (TPN) (38). In our study, preterm infants accounted for 25.0% of the cases, with a similar distribution between the genotypic positive and negative groups (27.6% vs. 22.2%, *P* = 0.642) as well as between the cholestatic and non-cholestatic groups (27.6% vs. 22.2%, *P* = 0.642). This balanced distribution suggests that preterm birth did not introduce significant bias to our genotype-phenotype association. TPN is a recognized risk factor for cholestatic jaundice, particularly in preterm infants requiring long-term parenteral nutrition (38). The lack of TPN data represents a limitation of our study, as it cannot exclude the possibility that certain cases of cholestatic jaundice, especially those without identifiable genetic causes, may be attributed to TPN. Future prospective studies should include detailed records of nutritional support to better elucidate the interactions between genetic predisposition and iatrogenic factors in the pathogenesis of infantile jaundice. Additionally, the total sample size was relatively small, with only 56 cases included, which to some extent restricted the statistical power and generalizability of the results. Particularly in subgroup analyses, such as investigating the association between specific gene mutation types (e.g., only 1 case of Dubin-Johnson syndrome) and clinical phenotypes, the insufficient sample size may prevent definitive conclusions. Due to the limited sample size, this study was unable to conduct more in-depth comparative analyses of cholestasis caused by different genetic etiologies, such as comparing clinical indicators between children with NTCP deficiency and Gilbert syndrome. Future studies should expand the sample size and conduct multicenter cohort studies to more accurately evaluate the clinical characteristics of neonatal jaundice under different genetic backgrounds and provide stronger evidence-based medical support for differential diagnosis.

In summary, this project utilized high-throughput next-generation sequencing technology to detect jaundice-related genes in infants with unexplained jaundice. Although there are many genes associated with jaundice, the common pathogenic genes mainly include the *UGT1A1* gene, *G6PD* gene, and *SLC10A1* gene, and these genes have high-frequency carrier sites in the population. Consequently, conducting gene sequencing on infants with unexplained jaundice is of considerable importance in clinical practice. In the future, screening for hot spots of common jaundice gene variations is expected to reduce costs and improve detection efficiency.

## Data Availability

The datasets presented in this study are included in the article and its Supplementary Material. Further inquiries can be directed to the corresponding author.

## References

[1] Bhutani VK Johnson L Sivieri EM. Predictive ability of a predischarge hour-specific serum bilirubin for subsequent significant hyperbilirubinemia in healthy term and near-term newborns. Pediatrics. (1999) 103(1):6–14. 10.1542/peds.103.1.6 9917432

[2] JianWu Q Min H Shiguang D. Research progress of hyperbilirubinemia effect on immune function in newborn. Med Recap. (2013) 19(20):3670–3. 10.3969/j.issn.1006-2084.2013.20.008

[3] Beibei M Qinghua W. Advances in research on the clinical phenotype and genetic etiology of jaundice associated with hereditary bilirubin metabolic disorders. Chin J Med Genet. (2023) 40(11):1436–40. 10.3760/cma.j.cn511374-20210223-00150 37906156

[4] Fawaz R Baumann U Ekong U Fischler B Hadzic N Mack CL Guideline for the evaluation of cholestatic jaundice in infants: joint recommendations of the north American society for pediatric gastroenterology, hepatology, and nutrition and the European society for pediatric gastroenterology, hepatology, and nutrition. J Pediatr Gastroenterol Nutr. (2017) 64(1):154–68. 10.1097/MPG.0000000000001334 27429428

[5] Richards S Aziz N Bale S Bick D Das S Gastier-Foster J Standards and guidelines for the interpretation of sequence variants: a joint consensus recommendation of the American college of medical genetics and genomics and the association for molecular pathology. Genet Med. (2015) 17(5):405–24. 10.1038/gim.2015.30 25741868 PMC4544753

[6] Karimzadeh P Fallahi M Kazemian M Taleghani NT Nouripour S Radfar M. Bilirubin induced encephalopathy. Iran J Child Neurol. (2020) 14(1):7–19. 10.22037/IJCN.V14I1.27890 32021624 PMC6956966

[7] ITOVA T. Climatic factors and prolonged neonatal jaundice. World J Biol Pharm Health Sci. (2023) 16(1):044–51. 10.30574/wjbphs.2023.16.1.0376

[8] Creeden JF Gordon DM Stec DE Hinds TD Jr. Bilirubin as a metabolic hormone: the physiological relevance of low levels. Am J Physiol Endocrinol Metaboli. (2021) 320(2):E191–207. 10.1152/ajpendo.00405.2020 PMC8260361 33284088

[9] Li R Gu X Wu G Deng Z Kang J Liang Z Genetic analysis in 331 cases of neonatal hyperbilirubinemia with unknown etiology. Chin J Neonatol. (2022) 37(06):520–4. 10.3760/cma.j.issn.2096-2932.2022.06.008

[10] Skierka JM Kotzer KE Lagerstedt SA O'Kane DJ Baudhuin LM. UGT1A1 Genetic analysis as a diagnostic aid for individuals with unconjugated hyperbilirubinemia. J Pediatr. (2013) 162(6):1146–52. 10.1016/j.jpeds.2012.11.042 23290513

[11] Akaba K Kimura T Sasaki A Tanabe S Ikegami T Hashimoto M Neonatal hyperbilirubinemia and mutation of the bilirubin uridine diphosphate-glucuronosyltransferase gene: a common missense mutation among Japanese, Koreans and Chinese. Iubmb Life. (1998) 46(1):21–6. 10.1080/15216549800203512 9784835

[12] Raijmakers MT Jansen PL Steegers EA Peters WH. Association of human liver bilirubin UDP-glucuronyltransferase activity with a polymorphism in the promoter region of the UGT1A1 gene. J Hepatol. (2000) 33(3):348–51. 10.1016/s0168-8278(00)80268-8 11019988

[13] Burchell B Hume R. Molecular genetic basis of gilbert’s syndrome. J Gastroenterol Hepatol. (1999) 14(10):960–6. 10.1046/j.1440-1746.1999.01984.x 10530490

[14] Kamisako T. What is Gilbert's syndrome? Lesson from genetic polymorphisms of UGT1A1 in Gilbert's syndrome from Asia. J Gastroenterol Hepatol. (2004) 19(9):955–7. 10.1111/j.1440-1746.2004.03524.x 15304109

[15] Teng HC Huang MJ Tang KS Yang SS Tseng CS Huang CS. Combined UGT1A1 and UGT1A7 variant alleles are associated with increased risk of Gilbert's syndrome in Taiwanese adults. Clin Genet. (2007) 72(4):321–8. 10.1111/j.1399-0004.2007.00873.x 17850628

[16] Lin R Wang X Wang Y Zhang F Wang Y Fu W Association of polymorphisms in four bilirubin metabolism genes with serum bilirubin in three Asian populations. Hum Mutat. (2009) 30(4):609–15. 10.1002/humu.20895 19243019

[17] Long J Zhang S Fang X Luo Y Liu J. Neonatal hyperbilirubinemia and Gly71Arg mutation of UGT1A1 gene: a Chinese case-control study followed by systematic review of existing evidence. Acta Paediatr. (2011) 100(7):966–71. 10.1111/j.1651-2227.2011.02176.x Erratum in: Acta Paediatr. 2012 Nov;101(11):1184. 21272068

[18] Beutler E Gelbart T Demina A. Racial variability in the UDP-glucuronosyltransferase 1 (UGT1A1) promoter: a balanced polymorphism for regulation of bilirubin metabolism? Proc Natl Acad Sci U S A. (1998) 95(14):8170–4. 10.1073/pnas.95.14.8170 9653159 PMC20948

[19] Bosma PJ Chowdhury JR Bakker C Gantla S de Boer A Oostra BA The genetic basis of the reduced expression of bilirubin UDP-glucuronosyltransferase 1 in Gilbert's syndrome. N Engl J Med. (1995) 333(18):1171–5. 10.1056/NEJM199511023331802 7565971

[20] Kaplan M Hammerman C Rubaltelli FF Vilei MT Levy-Lahad E Renbaum P Hemolysis and bilirubin conjugation in association with UDP-glucuronosyltransferase 1A1 promoter polymorphism. Hepatology. (2002) 35(4):905–11. 10.1053/jhep.2002.32526 11915038

[21] Tan YP Zhong DN Zhao K Xie XZ. Relationship between unexplained neonatal hyperbilirubinemia and UGTlAl gene mutation. J Guangxi Med Univ. (2022) 39(5):39. 10.16190/j.cnki.45-1211/r.2022.05.021

[22] Barateiro A Chen S Yueh M F Fernandes A Domingues HS Relvas J Reduced myelination and increased glia reactivity resulting from severe neonatal hyperbilirubinemia. Mol Pharmacol. (2016) 89(1):84–93. 10.1124/mol.115.098228 26480925 PMC4702100

[23] Vaz FM Paulusma CC Huidekoper H de Ru M Lim C Koster J Sodium taurocholate cotransporting polypeptide (SLC10A1) deficiency: conjugated hypercholanemia without a clear clinical phenotype. Hepatology. (2015) 61(1):260–7. 10.1002/hep.27240 24867799

[24] Qiu JW Deng M Cheng Y Atif RM Lin WX Guo L Sodium taurocholate cotransporting polypeptide (NTCP) deficiency: identification of a novel SLC10A1 mutation in two unrelated infants presenting with neonatal indirect hyperbilirubinemia and remarkable hypercholanemia. Oncotarget. (2017) 8(63):106598–607. 10.18632/oncotarget.22503 29290974 PMC5739759

[25] Deng LJ Ouyang WX Liu R Deng M Qiu JW Yaqub MR Clinical characterization of NTCP deficiency in paediatric patients: a case-control study based on SLC10A1 genotyping analysis. Liver Int. (2021) 41(11):2720–8. 10.1111/liv.15031 34369070 PMC9291912

[26] Ho RH Leake BF Roberts RL Lee W Kim RB. Ethnicity-dependent polymorphism in na+-taurocholate cotransporting polypeptide (SLC10A1) reveals a domain critical for bile acid substrate recognition. J Biol Chem. (2004) 279(8):7213–22. 10.1074/jbc.M305782200 14660639

[27] Pan W Song IS Shin HJ Kim MH Choi YL Lim SJ Genetic polymorphisms in na+-taurocholate co-transporting polypeptide (NTCP) and ileal apical sodium-dependent bile acid transporter (ASBT) and ethnic comparisons of functional variants of NTCP among Asian populations. Xenobiotica. (2011) 41(6):501–10. 10.3109/00498254.2011.555567 21341987

[28] Jiang W Yu G Liu P Geng Q Chen L Lin Q Structure and function of glucose-6-phosphate dehydrogenase-deficient variants in Chinese population. Hum Genet. (2006) 119(5):463–78. 10.1007/s00439-005-0126-5 16607506

[29] Boonyuen U Chamchoy K Swangsri T Saralamba N Day NP Imwong M. Detailed functional analysis of two clinical glucose-6-phosphate dehydrogenase (G6PD) variants, G6PDViangchan and G6PDViangchan + Mahidol: decreased stability and catalytic efficiency contribute to the clinical phenotype. Mol Genet Metab. (2016) 118(2):84–91. 10.1016/j.ymgme.2016.03.008 27053284 PMC4894296

[30] Beutler E Westwood B Kuhl W. Definition of the mutations of G6PD Wayne, G6PD Viangchan, G6PD Jammu, and G6PD “LeJeune’. Acta Haematol (1991) 86(4):179–82. 10.1159/000204830 1805484

[31] Nuchprayoon I Sanpavat S Nuchprayoon S. Glucose-6-phosphate dehydrogenase (G6PD) mutations in Thailand: G6PD Viangchan (871G>A) is the most common deficiency variant in the Thai population. Hum Mutat. (2002) 19(2):185. 10.1002/humu.9010 11793482

[32] Zhong YH Wu CH Chen GL Liu JF Qu YX. Correlation analysis between G6PD deficiency and pathological jaundice in neonates in northern Guangdong area. Chin J Birth Health & Heredity. (2016) 24(7):73–4. 10.13404/j.cnki.cjbhh.2016.07.031

[33] Wu H Zhu Q Zhong H Yu Z Zhang Q Huang Q. Analysis of genotype distribution of thalassemia and G6PD deficiency among Hakka population in Meizhou city of Guangdong province. J Clin Lab Anal. (2020) 34(4):e23140. 10.1002/jcla.23140 31793705 PMC7171329

[34] Zhong Z Wu H Li B Li C Liu Z Yang M Analysis of glucose-6-phosphate dehydrogenase genetic polymorphism in the Hakka population in southern China. Med Sci Monit. (2018) 24:7316–21. 10.12659/MSM.908402 30315739 PMC6196584

[35] Khan I Nawaz S Ullah I Saad M Khan MR Gul C Frequency, severity & outcome of G6PD deficiency among male newborns presenting to neonatal unit of A tertiary care hospital with neonatal jaundice. South East Euro J Public Health. (2024) XXV S1:2178–85. 10.70135/seejph.vi.2350

[36] Ayalew T Molla A Kefale B Alene TD Abebe GK Ngusie HS Factors associated with neonatal jaundice among neonates admitted at referral hospitals in northeast Ethiopia: a facility-based unmatched case-control study. BMC Pregnancy Childbirth. (2024) 24(1):150. 10.1186/s12884-024-06352-y 38383399 PMC10880319

[37] Narter F Can G Ergen A Isbir T Ince Z Çoban A. Neonatal hyperbilirubinemia and G71R mutation of the UGT1A1 gene in turkish patients. J Matern Fetal Neonatal Med. (2011) 24(2):313–6. 10.3109/14767058.2010.490889 20528217

[38] Wang N Yan W Hong L Lu L Feng Y Wu J Risk factors of parenteral nutrition-associated cholestasis in very-low-birthweight infants. J Paediatr Child Health. (2020) 56(11):1785–90. 10.1111/jpc.14826 32100397

