# Supplementary material for: Genetic insights into unexplained infant jaundice: a study from northern Guangdong, China
Source: Front Pediatr. 2026 Apr 13;14:1741740. doi: 10.3389/fped.2026.1741740 (PMC13111237; doi:10.3389/fped.2026.1741740)
Supplement: Supplementary file 1 [file Supplementaryfile1.docx]

Supplementary Material

# Supplementary Table: Classification of Genes and Variants by Pathogenic Mechanisms

## Supplementary Table 1. Overview of Gene Classification by Etiology

| **Category** | **Number of Genes** | **Percentage** |
| --- | --- | --- |
| Hemolysis-related | 32 | 19.9% |
| Bilirubin metabolism | 5 | 3.1% |
| Bile acid transport/synthesis | 20 | 12.4% |
| Hepatic dysfunction/Metabolic | 104 | 64.6% |
| **Total** | **161** | **100%** |

## Supplementary Table 2. Hemolysis-related - 32 genes

| **No.** | **Gene** | **RefSeq ID** | **Associated Diseases** | **Mechanism Subcategory** |
| --- | --- | --- | --- | --- |
| 1 | ADAMTS13 | NM_139025.4 | Schulman-Upshaw syndrome | Thrombotic thrombocytopenic purpura (TTP) |
| 2 | ALDOA | NM_184041.4 | Glycogen storage disease type XII (GSD XII) | Red cell aldolase deficiency |
| 3 | ANK1 | NM_000037.3 | Spherocytosis, hereditary, type 1 (HS1) | Hereditary spherocytosis |
| 4 | C15orf41 | NM_001130010.2 | Congenital dyserythropoietic anemia type Ib (CDA Ib) | Congenital erythropoietic disorders |
| 5 | CDAN1 | NM_138477.4 | Congenital dyserythropoietic anemia type I (CDA I) | Congenital erythropoietic disorders |
| 6 | COX4I2 | NM_032609.2 | Exocrine pancreatic insufficiency, dyserythropoietic anemia, and calvarial hyperostosis | Other hemolysis-related |
| 7 | EPB41 | NM_004437.3 | Spherocytosis, hereditary, type 1 (HS1) | Elliptocytosis, hereditary |
| 8 | G6PD | NM_001042351.2 | Hemolytic anemia, G6PD deficiency | Favism |
| 9 | GLRX5 | NM_016417.2 | Sideroblastic anemia, pyridoxine-refractory, autosomal recessive | Other hemolysis-related |
| 10 | GPI | NM_000175.4 | Glycosylphosphatidylinositol deficiency | Paroxysmal nocturnal hemoglobinuria (PNH) |
| 11 | GSR | NM_000637.5 | Glutathione reductase deficiency | Hemolytic anemia |
| 12 | HBA1 | NM_000558.4 | Alpha-thalassemia | Hemoglobin H disease |
| 13 | HBA2 | NM_000517.4 | Alpha-thalassemia | Hemoglobin H disease |
| 14 | HBB | NM_000518.4 | Beta-thalassemia | Sickle cell disease |
| 15 | HBG2 | NM_000184.2 | Hereditary persistence of fetal hemoglobin (HPFH) | Beta-thalassemia modifier |
| 16 | HK1 | NM_000188.2 | Hemolytic anemia due to hexokinase deficiency | Hexokinase deficiency |
| 17 | KCNN4 | NM_002250.2 | Gardos channelopathy | Dehydrated hereditary stomatocytosis (DHS) |
| 18 | KLF1 | NM_006563.4 | Congenital dyserythropoietic anemia type IV (CDA IV) | Hereditary persistence of fetal hemoglobin (HPFH) |
| 19 | LBR | NM_002296.3 | Pelger-Huët anomaly | Greenberg skeletal dysplasia |
| 20 | LYST | NM_000081.3 | Chédiak-Higashi syndrome (CHS) | Other hemolysis-related |
| 21 | PGK1 | NM_000291.3 | Phosphoglycerate kinase 1 deficiency (PGK1D) | Hemolytic anemia |
| 22 | PIEZO1 | NM_001142864.3 | Dehydrated hereditary stomatocytosis (DHS) | Lymphatic abnormalities |
| 23 | PKLR | NM_000298.5 | Pyruvate kinase deficiency (PKD) | Hemolytic anemia |
| 24 | PRF1 | NM_001083116.3 | Agranulocytosis, severe congenital, 4 (SCN4) | Lymphoma, non-Hodgkin |
| 25 | RHAG | NM_000324.2 | Overhydrated hereditary stomatocytosis (OHS) | Rh-null syndrome |
| 26 | RPS26 | NM_001029.5 | Diamond-Blackfan anemia 10 (DBA10) | Isolated congenital asplenia (ICA) |
| 27 | SEC23B | NM_006363.6 | Congenital dyserythropoietic anemia type II (CDA II) | HEMPAS |
| 28 | SLC4A1 | NM_000342.3 | Spherocytosis, hereditary, type 2 (HS2) | Elliptocytosis, hereditary |
| 29 | SPTA1 | NM_003126.4 | Elliptocytosis, hereditary, type 2 (HE2) | Pyropoikilocytosis, hereditary |
| 30 | SPTB | NM_001355436.2 | Spherocytosis, hereditary, type 2 (HS2) | Elliptocytosis, hereditary |
| 31 | TPI1 | NM_000365.5 | Triosephosphate isomerase deficiency | Hemolytic anemia |
| 32 | UROS | NM_000375.2 | Congenital erythropoietic porphyria (CEP) | Gunther disease |

## Supplementary Table 2. Bilirubin metabolism - 5 genes

| **No.** | **Gene** | **RefSeq ID** | **Associated Diseases** | **Mechanism Subcategory** |
| --- | --- | --- | --- | --- |
| 1 | ABCC2 | NM_000392.4 | Dubin-Johnson syndrome (DJS) | Hyperbilirubinemia, conjugated |
| 2 | BLVRA | NM_000712.3 | Hyperbiliverdinemia | Cholebilirubin reduction disorder |
| 3 | SLCO1B1 | NM_006446.4 | Hyperbilirubinemia, Rotor type | Statin-induced myopathy |
| 4 | SLCO1B3 | NM_019844.3 | Hyperbilirubinemia, Rotor type | Impaired bilirubin uptake |
| 5 | UGT1A1 | NM_000463.2 | Crigler-Najjar syndrome type I (CN1) | Crigler-Najjar syndrome type II (CN2) |

## Supplementary Table 2. **Bile acid transport/synthesis - 20 genes**

| **No.** | **Gene** | **RefSeq ID** | **Associated Diseases** | **Mechanism Subcategory** |
| --- | --- | --- | --- | --- |
| 1 | ABCB11 | NM_003742.2 | Progressive familial intrahepatic cholestasis type 2 (PFIC2) | Benign recurrent intrahepatic cholestasis type 2 (BRIC2) |
| 2 | ABCB4 | NM_000443.3 | Progressive familial intrahepatic cholestasis type 3 (PFIC3) | Intrahepatic cholestasis of pregnancy (ICP) |
| 3 | ACOX2 | NM_003500.3 | Bile acid synthesis defect, congenital, 6 (CBAS6) | Defect in bile acid synthase |
| 4 | AKR1D1 | NM_005989.3 | Bile acid synthesis defect, congenital, 2 (CBAS2) | Congenital bile acid synthesis defect |
| 5 | AMACR | NM_014324.5 | Bile acid synthesis defect, congenital, 4 (CBAS4) | Alpha-methylacyl-CoA racemase deficiency |
| 6 | ATP8B1 | NM_005603.6 | Progressive familial intrahepatic cholestasis type 1 (PFIC1) | Benign recurrent intrahepatic cholestasis type 1 (BRIC1) |
| 7 | BAAT | NM_001127610.1 | Bile acid-CoA:amino acid N-acyltransferase deficiency | Bile acid conjugation defect |
| 8 | CYP27A1 | NM_000784.3 | Cerebrotendinous xanthomatosis (CTX) | Cholestanol storage disease |
| 9 | CYP7B1 | NM_004820.4 | Spastic paraplegia 5A (SPG5A) | Congenital bile acid synthesis defect 3 (CBAS3) |
| 10 | HSD3B7 | NM_025193.3 | Bile acid synthesis defect, congenital, 3 (CBAS3) | Congenital bile acid synthesis defect |
| 11 | JAG1 | NM_000214.2 | Alagille syndrome 1 (ALGS1) | Tetralogy of Fallot |
| 12 | MYO5B | NM_001080467.2 | Microvillus inclusion disease (MVID) | Congenital enteropathy |
| 13 | NOTCH2 | NM_024408.3 | Alagille syndrome 2 (ALGS2) | Hajdu-Cheney syndrome |
| 14 | NR1H4 | NM_005123.3 | Familial intrahepatic cholestasis | Primary sclerosing cholangitis |
| 15 | SC5D | NM_006918.4 | Lathosterolosis | Cholestasis |
| 16 | SLC10A1 | NM_003049.3 | Sodium-taurocholate cotransporting polypeptide (NTCP) deficiency | Hypercholanemia, familial |
| 17 | SLC25A13 | NM_014251.2 | Citrin deficiency | Citrullinemia type II (CTLN2) |
| 18 | TJP2 | NM_004817.3 | Progressive familial intrahepatic cholestasis type 4 (PFIC4) | Hepatocellular carcinoma |
| 19 | VIPAS39 | NM_022067.3 | Arc syndrome 2 (VPS33B) | Arthrogryposis, renal dysfunction, and cholestasis 2 (ARC2) |
| 20 | VPS33B | NM_018668.4 | Arthrogryposis, renal dysfunction, and cholestasis 1 (ARC1) | Arc syndrome 1 |

## Supplementary Table 2.Hepatic dysfunction/Metabolic - 104 genes

| **No.** | **Gene** | **RefSeq ID** | **Associated Diseases** | **Mechanism Subcategory** |
| --- | --- | --- | --- | --- |
| 1 | ABCD3 | NM_002858.3 | Zellweger spectrum disorder | Peroxisome biogenesis disorder |
| 2 | ADK | NM_001123.3 | Adenosine kinase deficiency | Hypermethioninemia |
| 3 | AGL | NM_000642.2 | Glycogen storage disease type III (GSD III) | Cori disease |
| 4 | ALDOB | NM_000035.3 | Hereditary fructose intolerance (HFI) | Fructose metabolism disorder |
| 5 | ALG1 | NM_019109.4 | Congenital disorder of glycosylation type Ik (CDG-Ik) | congenital disorders of glycosylation(CDG) |
| 6 | ALG8 | NM_024079.4 | Congenital disorder of glycosylation type Ih (CDG-Ih) | congenital disorders of glycosylation(CDG) |
| 7 | AP1S1 | NM_001283.5 | Pettigrew syndrome | Mental retardation, X-linked, syndromic, 21 (MRXS21) |
| 8 | ARG1 | NM_000045.3 | Argininemia | Hyperargininemia |
| 9 | ASAH1 | NM_177924.4 | Farber lipogranulomatosis | Spinal muscular atrophy with progressive myoclonic epilepsy (SMA-PME) |
| 10 | ASL | NM_000048.3 | Argininosuccinate lyase deficiency | Argininosuccinic aciduria |
| 11 | ASS1 | NM_000050.4 | Citrullinemia type I (CTLN1) | Argininosuccinate synthetase deficiency |
| 12 | ATP11C | NM_001010986.2 | Immunodeficiency 33 (IMD33) | B cell defect |
| 13 | ATP6AP1 | NM_001183.5 | Immunodeficiency 47 (IMD47) | X-linked immunodeficiency |
| 14 | ATP6AP2 | NM_005765.2 | Renal tubular dysgenesis (RTD) | Mental retardation, X-linked, with epilepsy |
| 15 | ATP7B | NM_000053.3 | Wilson disease (WD) | Hepatolenticular degeneration |
| 16 | BCS1L | NM_004328.4 | Mitochondrial complex III deficiency, nuclear type 1 (MC3DN1) | Björnstad syndrome |
| 17 | CC2D2A | NM_001080522.2 | Meckel syndrome type 8 (MKS8) | Joubert syndrome 9 (JBTS9) |
| 18 | CCDC115 | NM_032357.3 | Congenital disorder of glycosylation type IIo (CDG-IIo) | congenital disorders of glycosylation(CDG) |
| 19 | CFTR | NM_000492.3 | Cystic fibrosis (CF) | Congenital bilateral absence of vas deferens (CBAVD) |
| 20 | CLDN1 | NM_021101.4 | Nonsyndromic deafness | Ichthyosis, leukocyte vacuoles, alopecia, and sclerosing cholangitis (ILVASC) |
| 21 | COG4 | NM_015386.2 | Congenital disorder of glycosylation type IIj (CDG-IIj) | congenital disorders of glycosylation(CDG) |
| 22 | COG6 | NM_020751.2 | Congenital disorder of glycosylation type IIl (CDG-IIl) | congenital disorders of glycosylation(CDG) |
| 23 | COG7 | NM_153603.3 | Congenital disorder of glycosylation type IIe (CDG-IIe) | congenital disorders of glycosylation(CDG) |
| 24 | CPT1A | NM_001876.3 | Carnitine palmitoyltransferase 1A deficiency (CPT1A deficiency) | Hepatic encephalopathy |
| 25 | DCDC2 | NM_016356.4 | Nephronophthisis 19 (NPHP19) | Molar tooth malformation |
| 26 | DGUOK | NM_080916.2 | Mitochondrial DNA depletion syndrome 3 (MTDPS3) | Hepatocerebral mitochondrial DNA depletion syndrome |
| 27 | DHCR7 | NM_001360.2 | Smith-Lemli-Opitz syndrome (SLOS) | 7-dehydrocholesterol reductase deficiency |
| 28 | DPAGT1 | NM_001382.3 | Congenital disorder of glycosylation type Ij (CDG-Ij) | Myasthenic syndrome, congenital, 13 (CMS13) |
| 29 | ETFA | NM_000126.3 | Glutaric acidemia IIA (GAIIA) | Multiple acyl-CoA dehydrogenase deficiency (MADD) |
| 30 | ETFB | NM_001985.2 | Glutaric acidemia IIB (GAIIB) | Multiple acyl-CoA dehydrogenase deficiency (MADD) |
| 31 | ETFDH | NM_004453.3 | Glutaric acidemia IIC (GAIIC) | Multiple acyl-CoA dehydrogenase deficiency (MADD) |
| 32 | FAH | NM_000137.2 | Tyrosinemia type I (TYRSN1) | Hepatorenal tyrosinemia |
| 33 | FBP1 | NM_000507.3 | Fructose-1,6-bisphosphatase deficiency | Hypoglycemia |
| 34 | FH | NM_000143.3 | Fumarase deficiency | Fumaric aciduria |
| 35 | G6PC | NM_000151.3 | Glycogen storage disease type Ia (GSD Ia) | von Gierke disease |
| 36 | GAA | NM_000152.4 | Glycogen storage disease type II (GSD II) | Pompe disease |
| 37 | GALE | NM_000403.3 | Epimerase-deficiency galactosemia (GALE deficiency) | Other metabolic liver diseases |
| 38 | GALT | NM_000155.3 | Galactosemia (GALT deficiency) | Classical galactosemia |
| 39 | GBA | NM_001005741.2 | Gaucher disease type I | Gaucher disease type II |
| 40 | GBE1 | NM_000158.3 | Glycogen storage disease type IV (GSD IV) | Andersen disease |
| 41 | GFM1 | NM_024996.5 | Combined oxidative phosphorylation deficiency 1 (COXPD1) | Hepatoencephalopathy |
| 42 | GLIS3 | NM_152629.3 | Neonatal diabetes mellitus with congenital hypothyroidism (NDH) | Diabetes mellitus, permanent neonatal 1 (PNDM1) |
| 43 | GUSB | NM_000181.3 | Mucopolysaccharidosis type VII (MPS VII) | Sly syndrome |
| 44 | HADHA | NM_000182.4 | Long-chain 3-hydroxyacyl-CoA dehydrogenase deficiency (LCHAD deficiency) | Mitochondrial trifunctional protein deficiency (MTPD) |
| 45 | HSD17B4 | NM_000414.3 | D-bifunctional protein deficiency | Peroxisomal bifunctional enzyme deficiency |
| 46 | IARS | NM_002161.5 | Growth retardation, intellectual developmental disorder, hypotonia, and hepatopathy (GIDH) | Amino acid tRNA synthetase deficiency |
| 47 | IGSF1 | NM_001170961.1 | X-linked central hypothyroidism with testicular enlargement (XCHT) | Hypothyroidism, central |
| 48 | INSR | NM_000208.3 | Insulin resistance syndrome | Donohue syndrome (leprechaunism) |
| 49 | KMT2D | NM_003482.3 | Kabuki syndrome 1 (KABUK1) | Other metabolic liver diseases |
| 50 | KRT18 | NM_000224.2 | Cryptogenic cirrhosis | Liver disease |
| 51 | KRT8 | NM_002273.3 | Cryptogenic cirrhosis | Liver disease |
| 52 | LARS1 | NM_020117.10 | Infantile liver failure syndrome 1 (ILFS1) | Other metabolic liver diseases |
| 53 | LIPA | NM_000235.3 | Wolman disease | Cholesteryl ester storage disease (CESD) |
| 54 | LIPT1 | NM_145199.2 | Lipoic acid biosynthesis defect | Sulfuric acid synthesis disorder |
| 55 | LPL | NM_000237.2 | Lipoprotein lipase deficiency (LPLD) | Hyperlipoproteinemia type I |
| 56 | MARS1 | NM_004990.3 | Interstitial lung and liver disease (ILLD) | Pulmonary alveolar proteinosis |
| 57 | MPI | NM_002435.2 | Congenital disorder of glycosylation type Ib (CDG-Ib) | Mannose-6-phosphate isomerase deficiency |
| 58 | MPV17 | NM_002437.4 | Mitochondrial DNA depletion syndrome 6 (MTDPS6) | Navajo neurohepatopathy (NNH) |
| 59 | MTR | NM_000254.2 | Methylmalonic aciduria and homocystinuria, cblG type | Homocystinuria-megaloblastic anemia, cblG complementation type |
| 60 | NBAS | NM_015909.3 | Short stature, optic nerve atrophy, and Pelger-Huët anomaly (SOPH) | Infantile liver failure syndrome 2 (ILFS2) |
| 61 | NEK8 | NM_178170.2 | Nephronophthisis 9 (NPHP9) | Renal-hepatic-pancreatic dysplasia 2 (RHPD2) |
| 62 | NPC1 | NM_000271.4 | Niemann-Pick disease type C1 (NPC1) | Obesity, susceptibility to |
| 63 | NPC2 | NM_006432.3 | Niemann-Pick disease type C2 (NPC2) | lysosomal storage disease |
| 64 | NPHP3 | NM_153240.4 | Nephronophthisis 3 (NPHP3) | Renal-hepatic-pancreatic dysplasia |
| 65 | PEPD | NM_000285.3 | Prolidase deficiency | Iminodipeptiduria |
| 66 | PEX1 | NM_000466.2 | Zellweger spectrum disorder (ZSD) | Peroxisome biogenesis disorder 1A (PBD1A) |
| 67 | PEX12 | NM_000286.2 | Zellweger spectrum disorder (ZSD) | Peroxisome biogenesis disorder 3A (PBD3A) |
| 68 | PEX13 | NM_002618.3 | Zellweger spectrum disorder (ZSD) | Peroxisome biogenesis disorder 11A (PBD11A) |
| 69 | PEX14 | NM_004565.2 | Zellweger spectrum disorder (ZSD) | Peroxisomal diseases |
| 70 | PEX16 | NM_004813.2 | Zellweger spectrum disorder (ZSD) | Peroxisome biogenesis disorder 8A (PBD8A) |
| 71 | PEX19 | NM_002857.3 | Zellweger spectrum disorder (ZSD) | Peroxisome biogenesis disorder 12A (PBD12A) |
| 72 | PEX2 | NM_000318.2 | Zellweger spectrum disorder (ZSD) | Peroxisome biogenesis disorder 5A (PBD5A) |
| 73 | PEX26 | NM_017929.5 | Zellweger spectrum disorder (ZSD) | Peroxisome biogenesis disorder 7B (PBD7B) |
| 74 | PEX3 | NM_003630.2 | Zellweger spectrum disorder (ZSD) | Peroxisome biogenesis disorder 10A (PBD10A) |
| 75 | PEX5 | NM_001131025.1 | Zellweger spectrum disorder (ZSD) | Peroxisome biogenesis disorder 2A (PBD2A) |
| 76 | PEX6 | NM_000287.3 | Zellweger spectrum disorder (ZSD) | Peroxisome biogenesis disorder 4A (PBD4A) |
| 77 | PFKM | NM_000289.5 | Glycogen storage disease type VII (GSD VII) | Tarui disease |
| 78 | PHKA2 | NM_000292.2 | Glycogen storage disease type IXa (GSD IXa) | Liver phosphorylase kinase deficiency |
| 79 | PHKB | NM_000293.2 | Glycogen storage disease type IXb (GSD IXb) | Pompe's syndrome |
| 80 | PHKG2 | NM_000294.2 | Glycogen storage disease type IXc (GSD IXc) | Liver phosphorylase kinase deficiency |
| 81 | POLG | NM_002693.2 | Progressive external ophthalmoplegia (PEO) | Mitochondrial DNA depletion syndrome 4A (MTDPS4A) |
| 82 | POLG2 | NM_007215.3 | Progressive external ophthalmoplegia 3 (PEO3) | Mitochondrial DNA depletion syndrome |
| 83 | POMC | NM_001035256.2 | Obesity, early-onset, with adrenal insufficiency and red hair | Adrenal insufficiency |
| 84 | POU1F1 | NM_000306.3 | Combined pituitary hormone deficiency 1 (CPHD1) | Pituitary dwarfism |
| 85 | PYGL | NM_002863.4 | Glycogen storage disease type VI (GSD VI) | Hers disease |
| 86 | RFX6 | NM_173560.3 | Mitchell-Riley syndrome | Diabetes, neonatal, with pancreatic hypoplasia |
| 87 | RINT1 | NM_021930.5 | Breast cancer, susceptibility to | tumor susceptibility gene |
| 88 | RPGRIP1L | NM_015272.4 | Meckel syndrome type 5 (MKS5) | Joubert syndrome 7 (JBTS7) |
| 89 | SCYL1 | NM_020680.3 | Liver failure, acute infantile | Spinocerebellar ataxia |
| 90 | SERAC1 | NM_032861.3 | 3-methylglutaconic aciduria with deafness, encephalopathy, and Leigh-like syndrome (MEGDEL) | Methylenediamine glycol(MEGDEL) |
| 91 | SERPINA1 | NM_000295.4 | Alpha-1-antitrypsin deficiency (AATD) | Emphysema |
| 92 | SLC17A5 | NM_012434.4 | Salla disease | Infantile free sialic acid storage disease (ISSD) |
| 93 | SLC25A15 | NM_014252.3 | Hyperornithinemia-hyperammonemia-homocitrullinuria syndrome (HHH syndrome) | Defects in solute transport proteins |
| 94 | SLC2A1 | NM_006516.2 | Glucose transporter type 1 deficiency syndrome (GLUT1DS) | Infantile seizures |
| 95 | SLC30A10 | NM_018713.2 | Hypermanganesemia with dystonia, polycythemia, and cirrhosis (HMDPC) | Manganese metabolic disorder |
| 96 | SLC37A4 | NM_001164277.1 | Glycogen storage disease type Ib (GSD Ib) | Neutropenia |
| 97 | SMPD1 | NM_000543.4 | Niemann-Pick disease type A (NPA) | Niemann-Pick disease type B (NPB) |
| 98 | TBX19 | NM_005149.2 | Adrenal insufficiency, congenital, with ACTH deficiency | Isolated ACTH deficiency |
| 99 | TFAM | NM_003201.2 | Mitochondrial DNA depletion syndrome | Mitochondrial disease |
| 100 | TMEM67 | NM_153704.5 | Meckel syndrome type 3 (MKS3) | Nephronophthisis 11 (NPHP11) |
| 101 | TRMU | NM_018006.4 | Liver failure, infantile | Mitochondrial disease |
| 102 | TTC37 | NM_014639.3 | Trichohepatoenteric syndrome 1 (THES1) | Syndromic diarrhea |
| 103 | TWNK | NM_021830.4 | Progressive external ophthalmoplegia with mitochondrial DNA deletions 3 (PEOA3) | Infantile-onset spinocerebellar ataxia (IOSCA) |
| 104 | VHL | NM_000551.3 | von Hippel-Lindau disease (VHL) | Renal cell carcinoma |
